# Supplementary material for: GWAS identifies 14 loci for device-measured physical activity and sleep duration
Source: Nat Commun. 2018 Dec 10;9:5257. doi: 10.1038/s41467-018-07743-4 (PMC6288145; doi:10.1038/s41467-018-07743-4)

***GWAS identifies 14 loci for device-measured physical activity and sleep duration*.**

Doherty et al.

Supplementary Figure 1 | Phenotype face validity check: difference in accelerometer-measured sleep duration classification by self-reported chronotype in 91,105 UK Biobank participants.

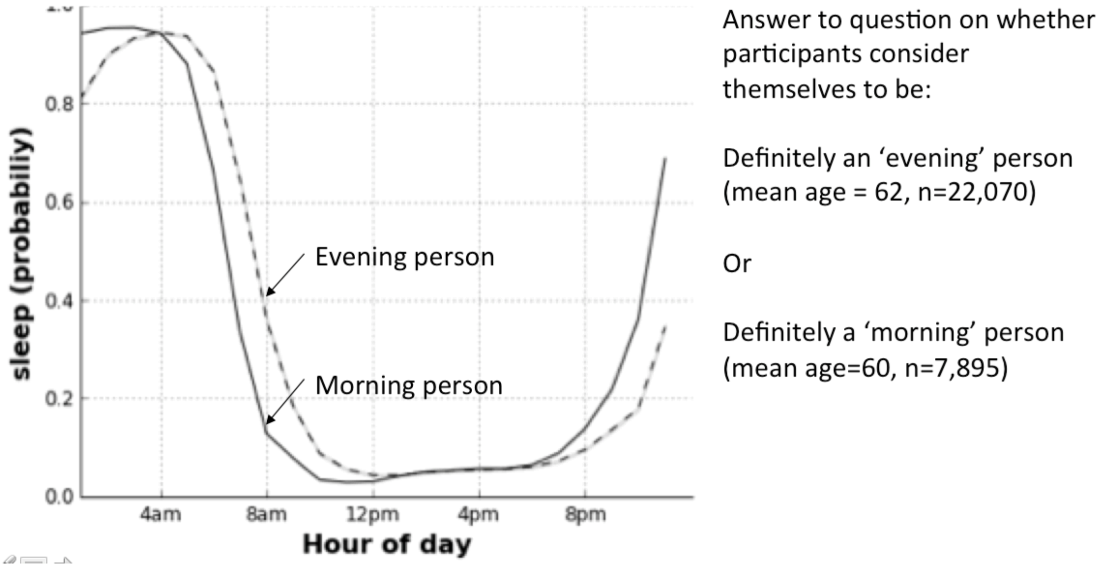

Supplementary Figure 2 | Phenotypic and genetic correlation between traditional 'overall activity' metric and four machine-learned phenotypes in 91,105 UK Biobank participants who wore wrist-worn accelerometers.

Phenotypic correlations

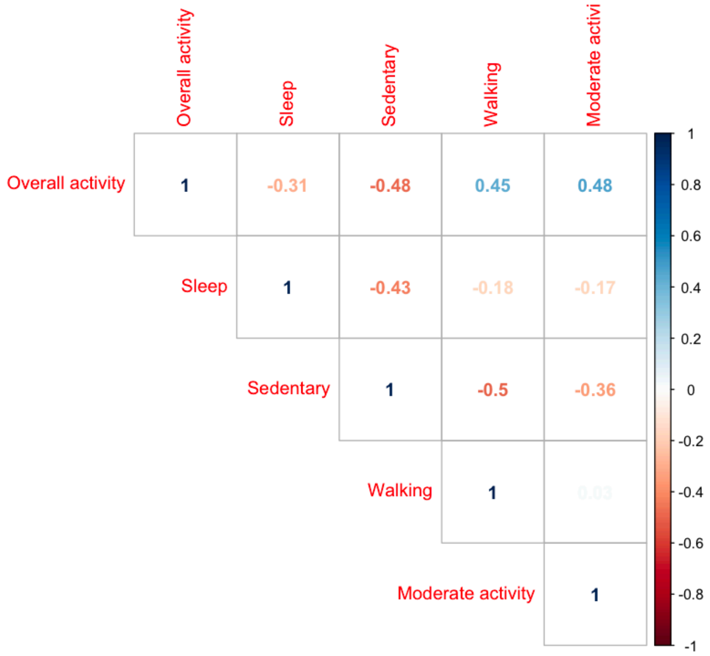

Genetic correlations

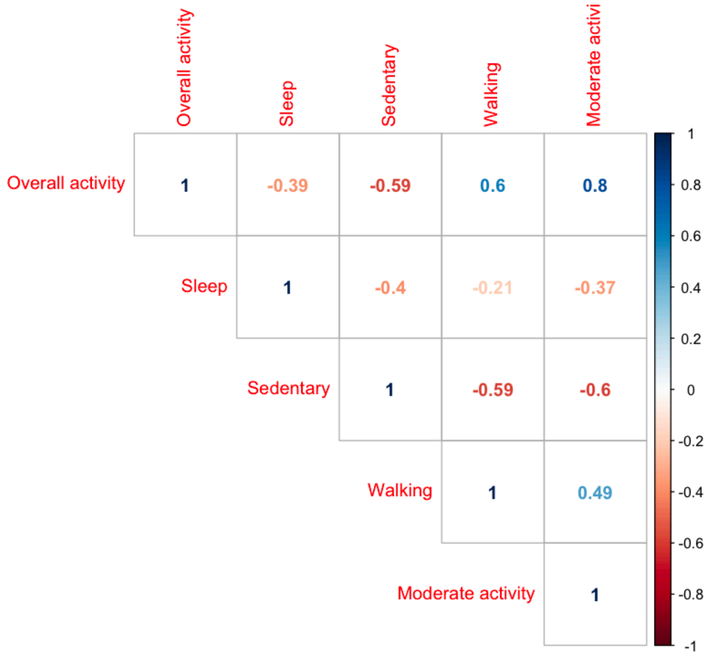

Supplementary Figure 3 | Summary Manhattan and QQ plots of European sex-combined GWAS of accelerometer-measured physical activity and sleep duration traits: the UK Biobank study 2013-2015 (n = 91,122).
Genomic control (λ), explained variance (R^2) and heritability (h^2) estimates are also provided.

Overall activity*:* $\lambda = 1.20$, LD intercept = 1.01, $h^2 = 0.21$,
R^2(GWS loci) = 0.06%, R^2($5x10^{-3}$ loci) = 14.0%

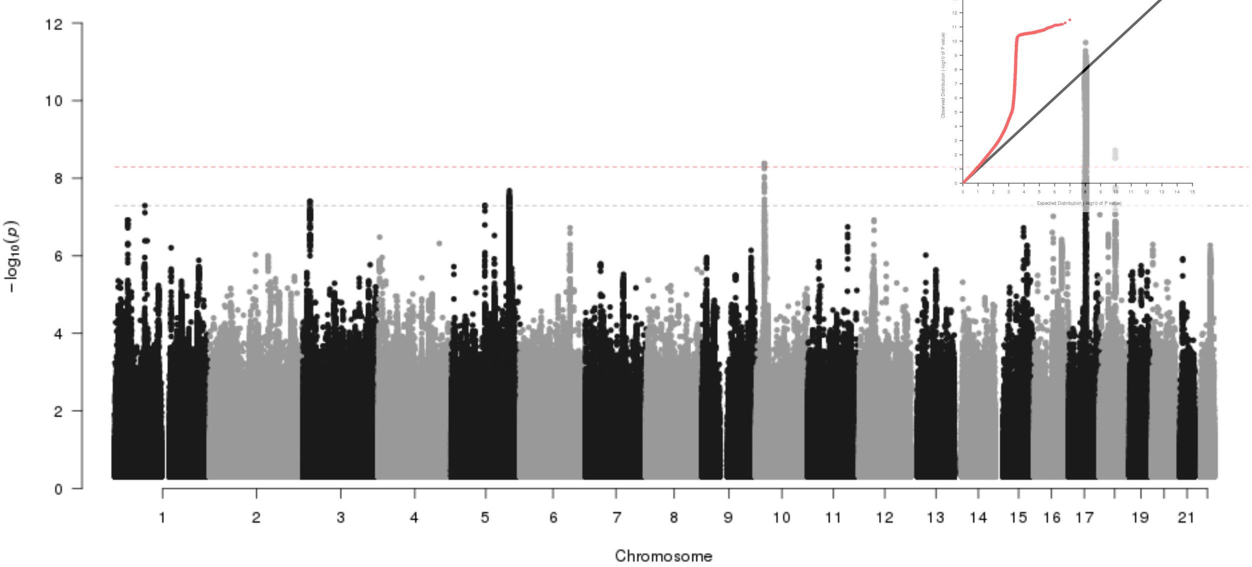

Sleep duration*:* $\lambda = 1.20$, LD intercept = 1.01, $h^2 = 0.19$,
R^2(GWS loci) = 0.33%, R^2($5x10^{-3}$ loci) = 18.1%

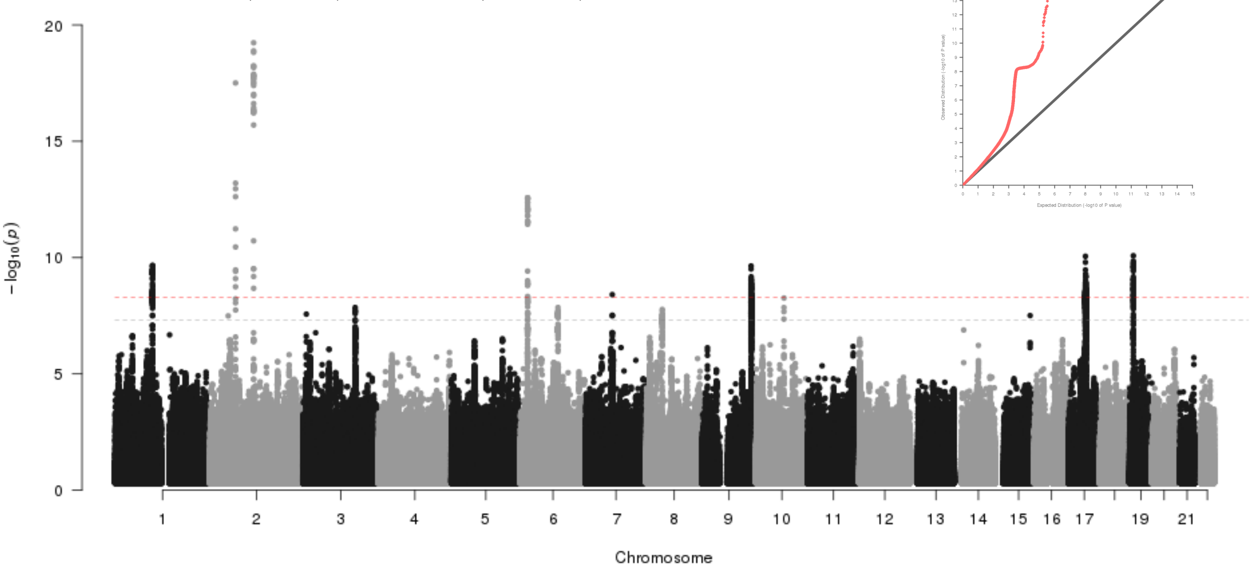

Sedentary*:* $\lambda = 1.20$, LD intercept = 0.98, $h^2 = 0.15$,
R^2(GWS loci) = 0.08%, R^2($5x10^{-3}$ loci) = 12.9%

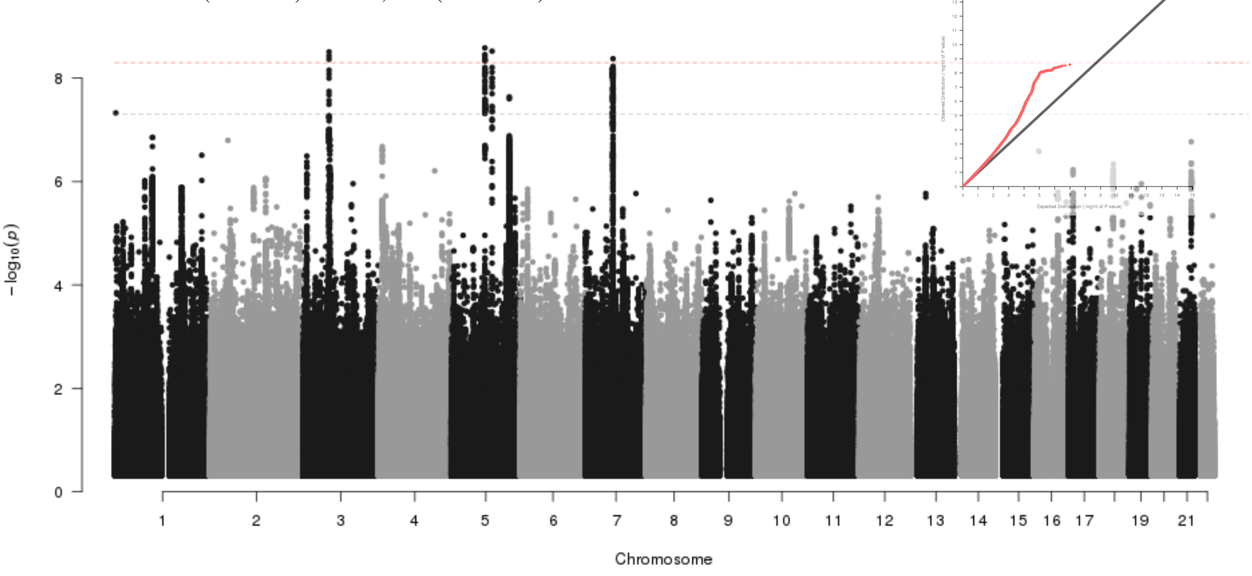

Walking: $\lambda = 1.15$, LD intercept = 1.00, $h^2 = 0.12$,
R^2(GWS loci) = nan%, $R^2(5\text{x}10^{-3}$ loci) = 16.4%

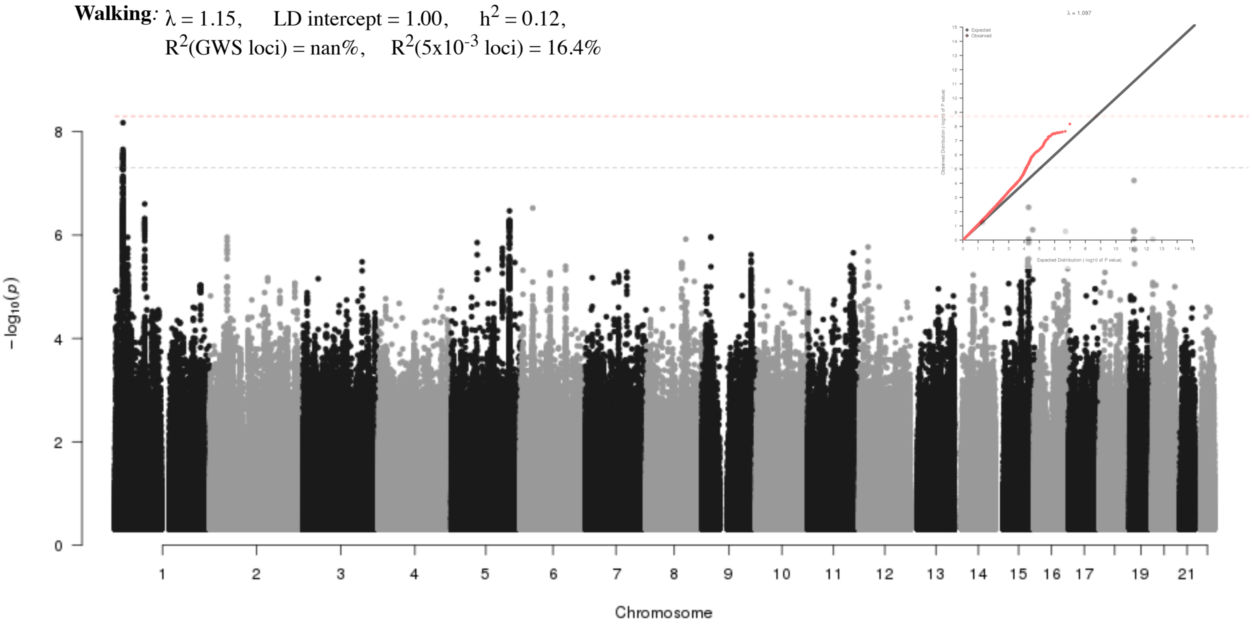

Moderate intensity activity: $\lambda = 1.10$, LD intercept = 1.02, $h^2 = 0.10$,
R^2(GWS loci) = nan%, $R^2(5\text{x}10^{-3}$ loci) = 14.5%

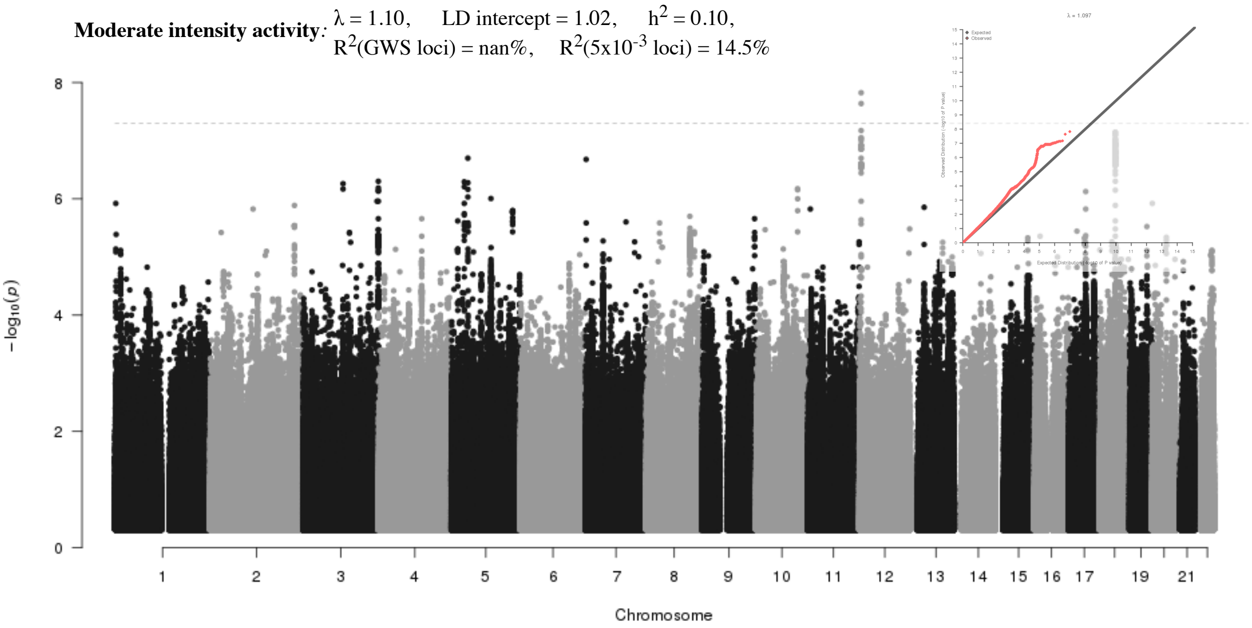

Supplementary Figure 4 | Regional association plots for genome-wide significant loci associated with accelerometer-measured physical activity and sleep duration traits in 91,105 UK Biobank participants.
A. Regional plots for novel loci. B. Regional plots for previously identified loci. Nearest gene is labelled at the top of each plot.

Overall activity

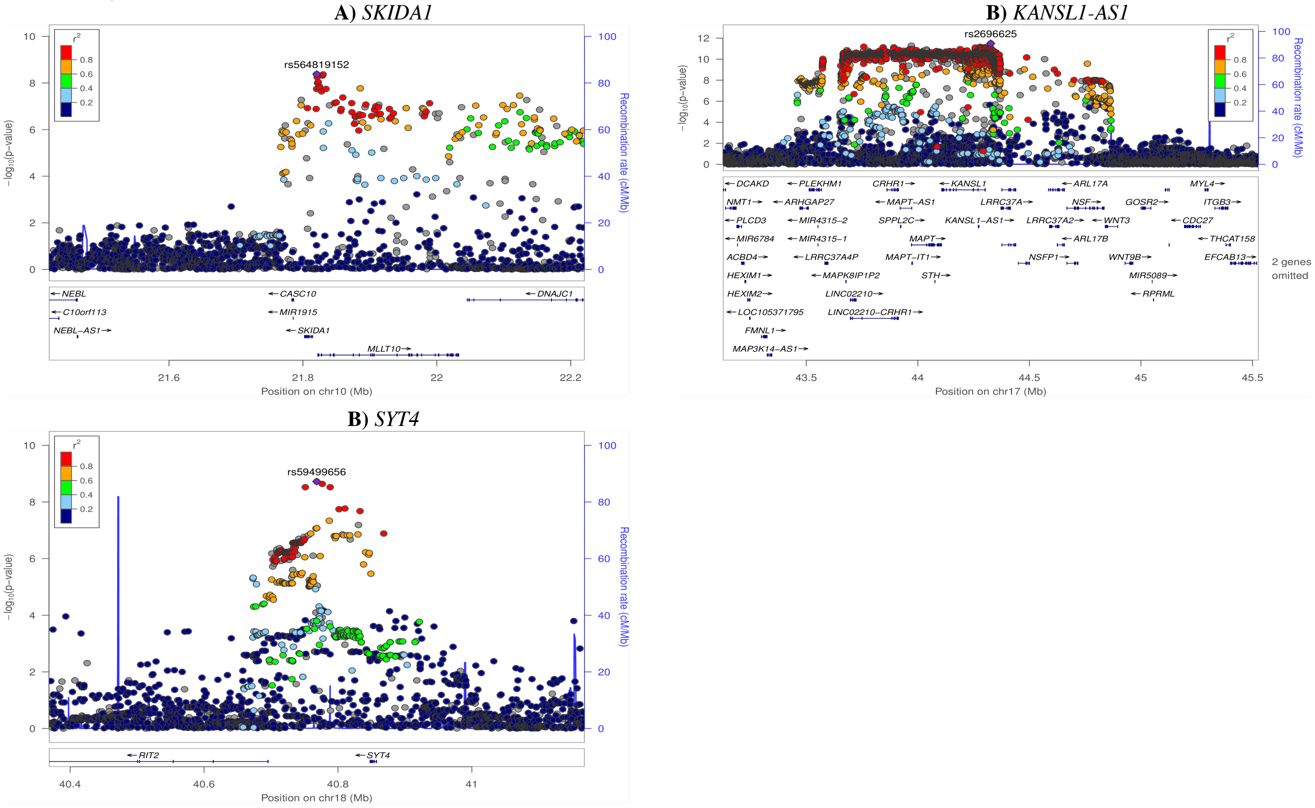

Sleep duration

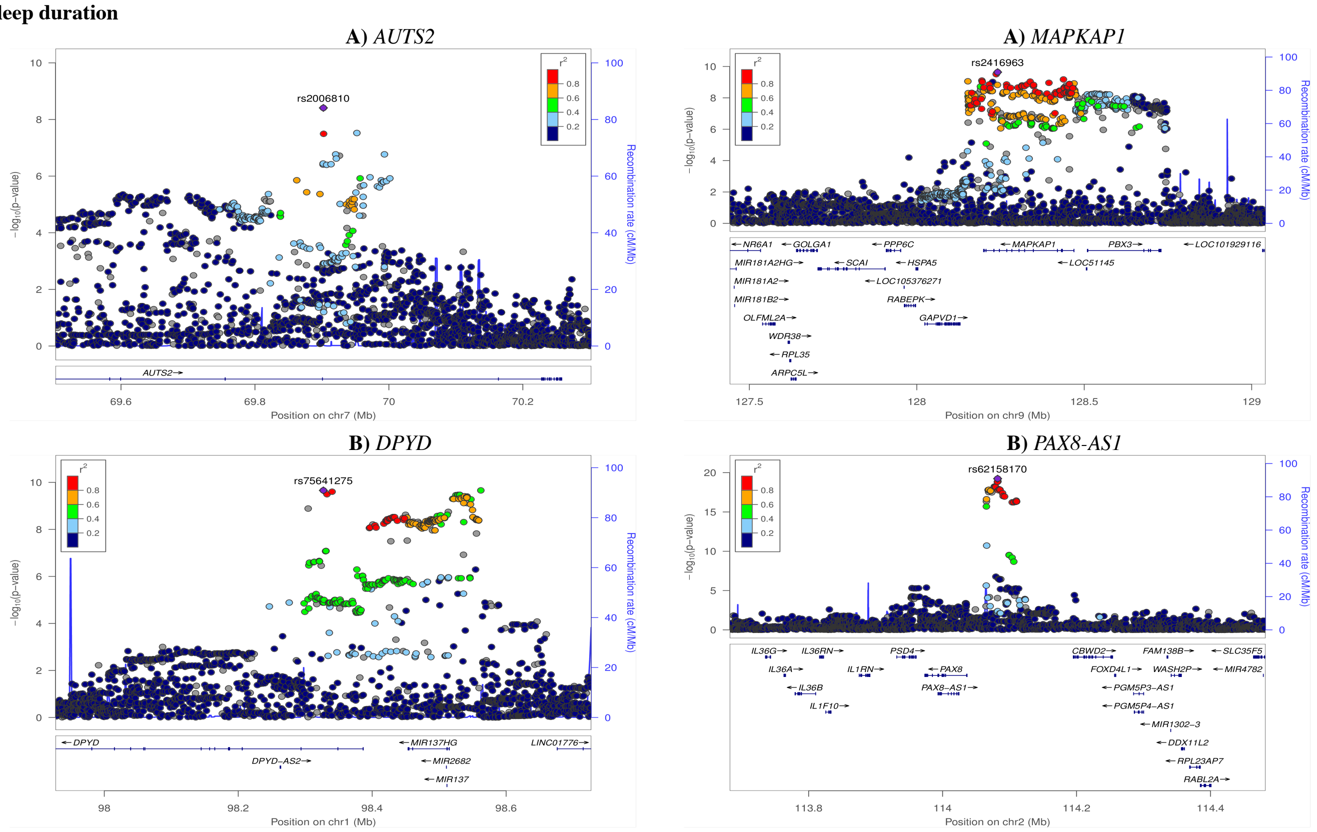

B) *MEIS1*

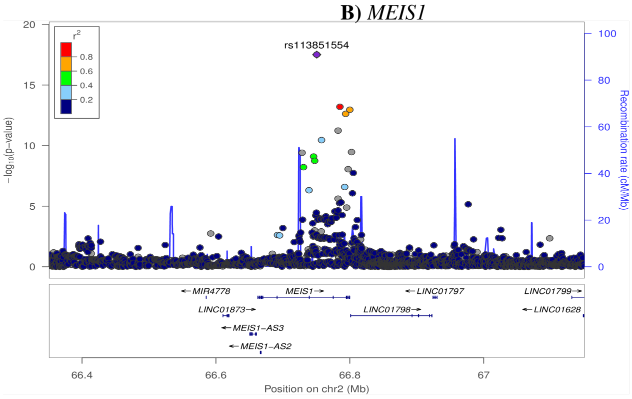

B) *LOC101928519*

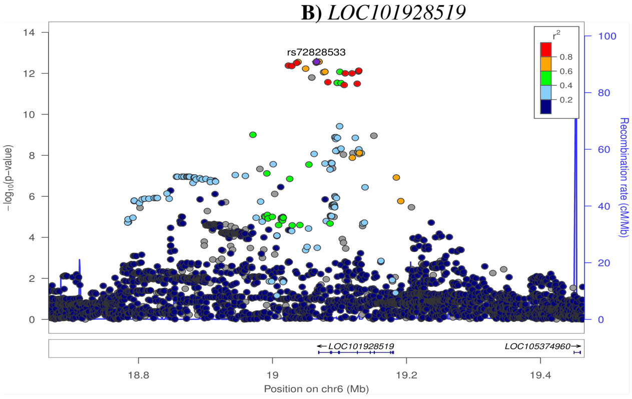

B) *MAPK8IP1P2*

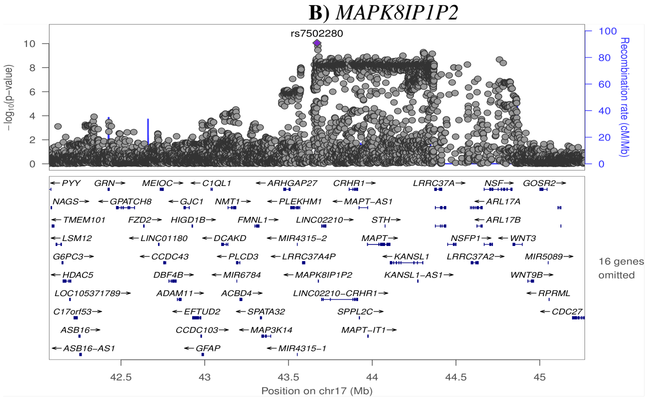

B) *OLFM2*

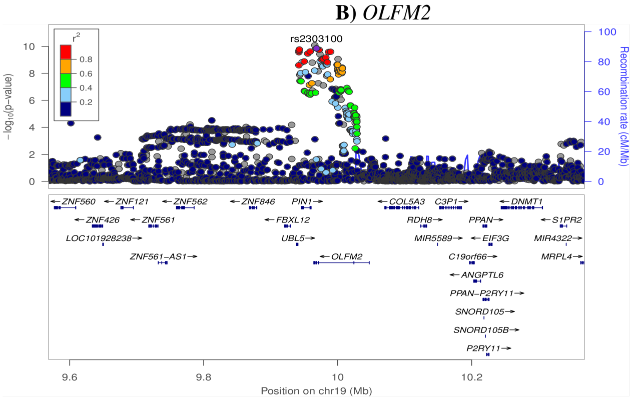

Sedentary

A) *LOC105377146*

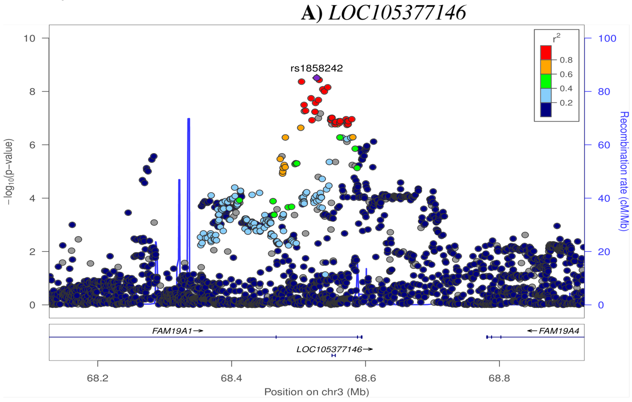

A) *MEF2C-AS2*

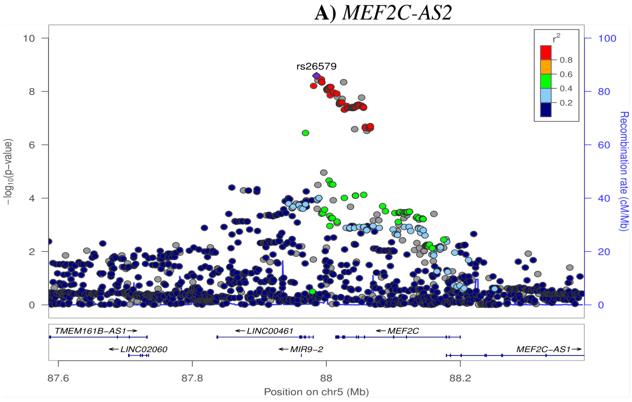

A) *EFNA5*

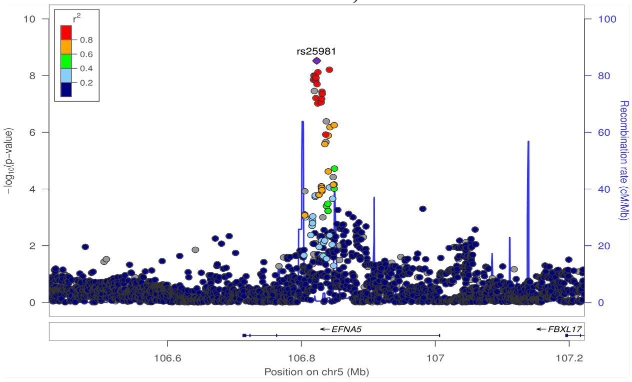

A) *CALN1*

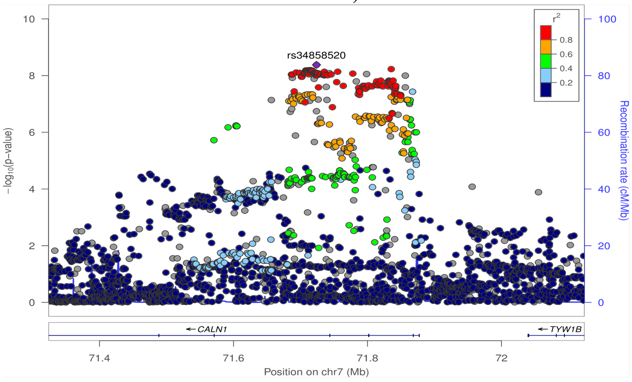

Supplementary Figure 5 | Heritability partitioning enrichment estimates across functional categories and tissues for accelerometer-measured physical activity and sleep duration traits in 91,105 UK Biobank participants.
Error bars represent 95% confidence intervals around the estimate, proportion of SNPs indicated in x-axis label parentheses. Annotated categories indicate annotations or tissues that pass the multiple testing significance threshold ($p<4.76x10^{-4}$ for functional annotations, $p<1x10^{-3}$ for cell types).

Overall activity

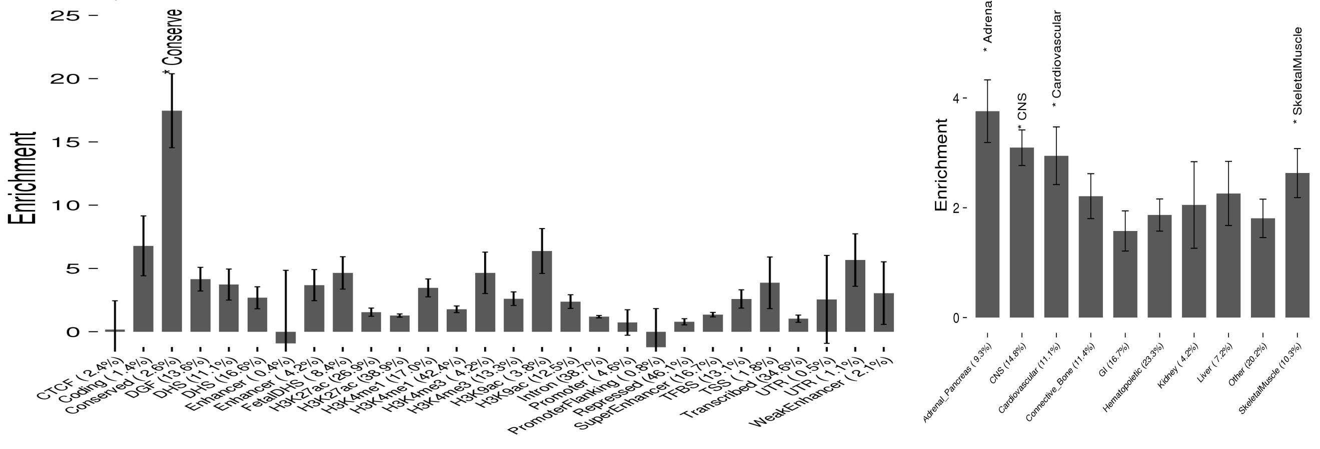

Sleep duration

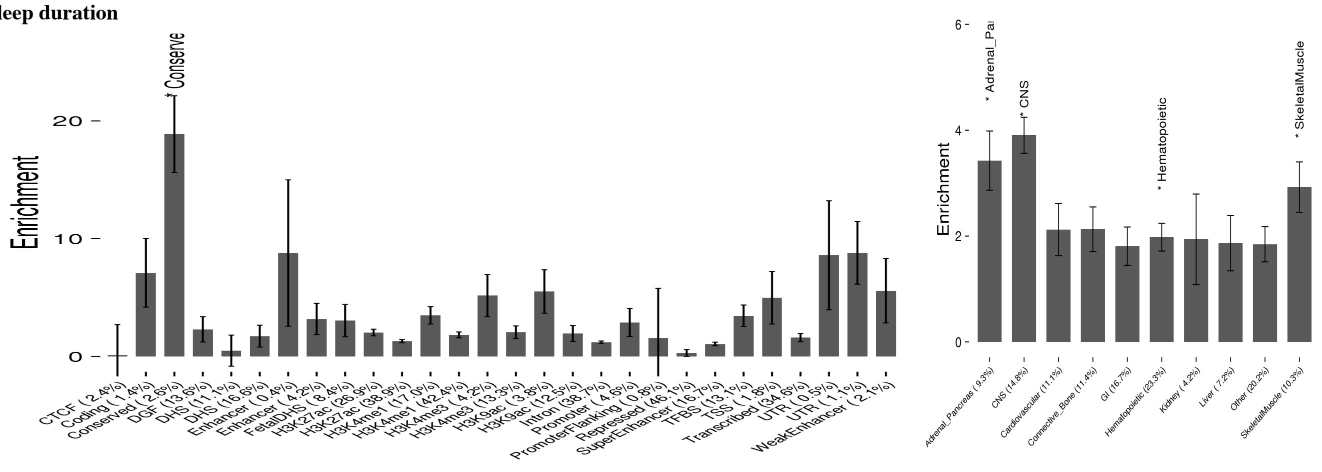

Sedentary

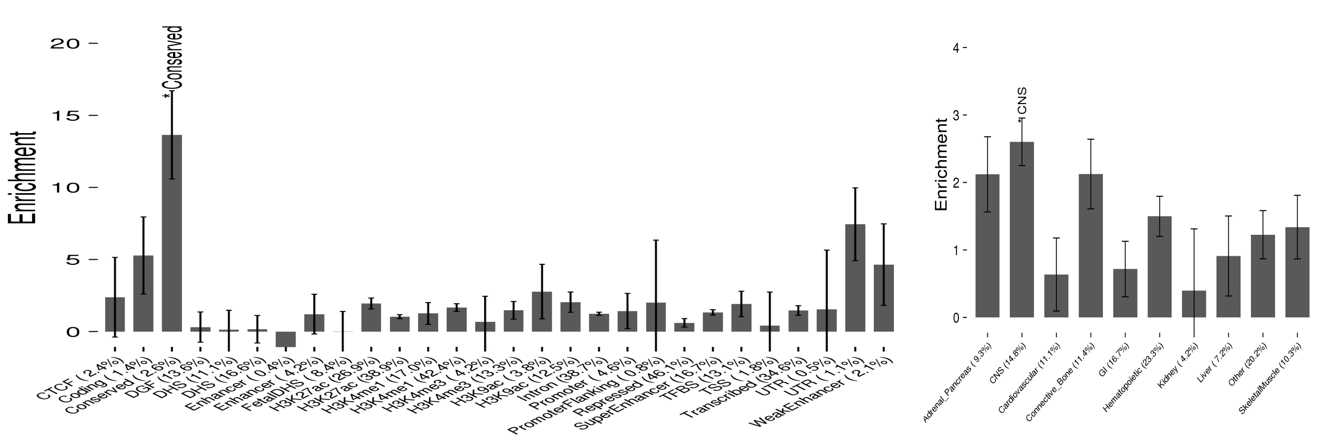

Walking

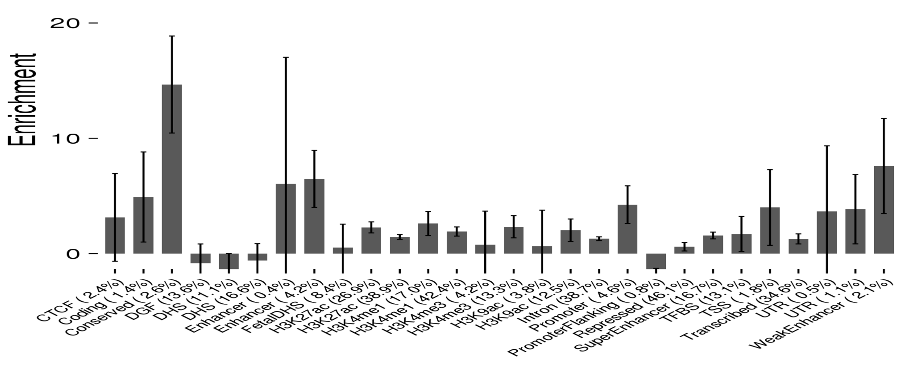

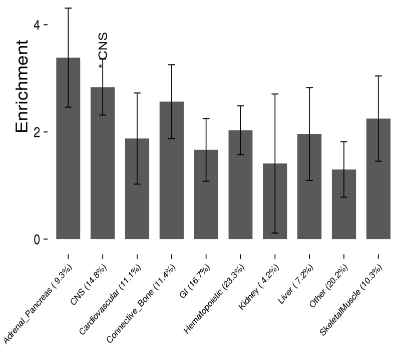

Moderate intensity activity

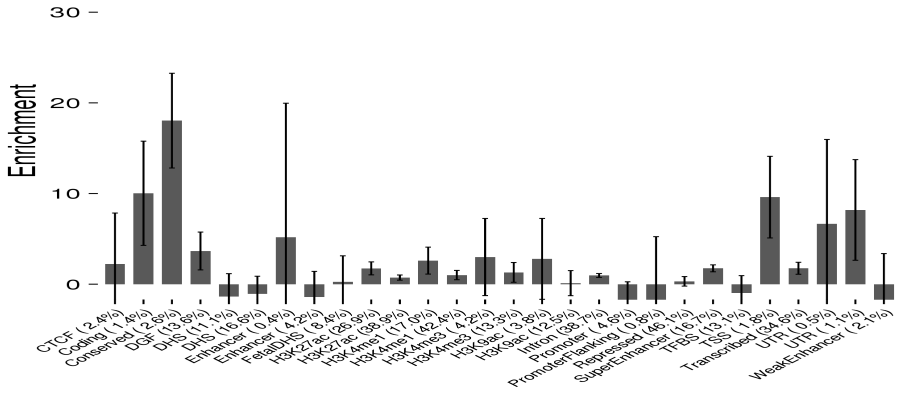

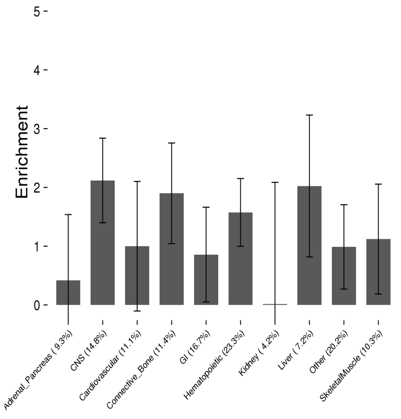

Supplementary Figure 6 | Summary Miami and QQ plots of European sex-specific analysis of accelerometer-measured physical activity and sleep duration traits (39,968 men, 51,137 women).
Miami plot of association statistics (-log10 (P values)) for women on the positive y-axis and men on the negative y-axis. Quantile-quantile plot of SNP associations stratified by sex. Genomic control (λ), explained variance (R^2) and heritability (h^2) estimates are also provided.

Overall activity: λ (m) = 1.1, LD α (m) = 1.0, h^2 (m) = 0.2, R^2(5x10^{-7}hits m) = 0.0%
Rg = 0.98 λ (w)= 1.15, LD α (w) = 1.02, h^2 (w) = 0.23 R^2(5x10^{-7}hits f) = 0.03%

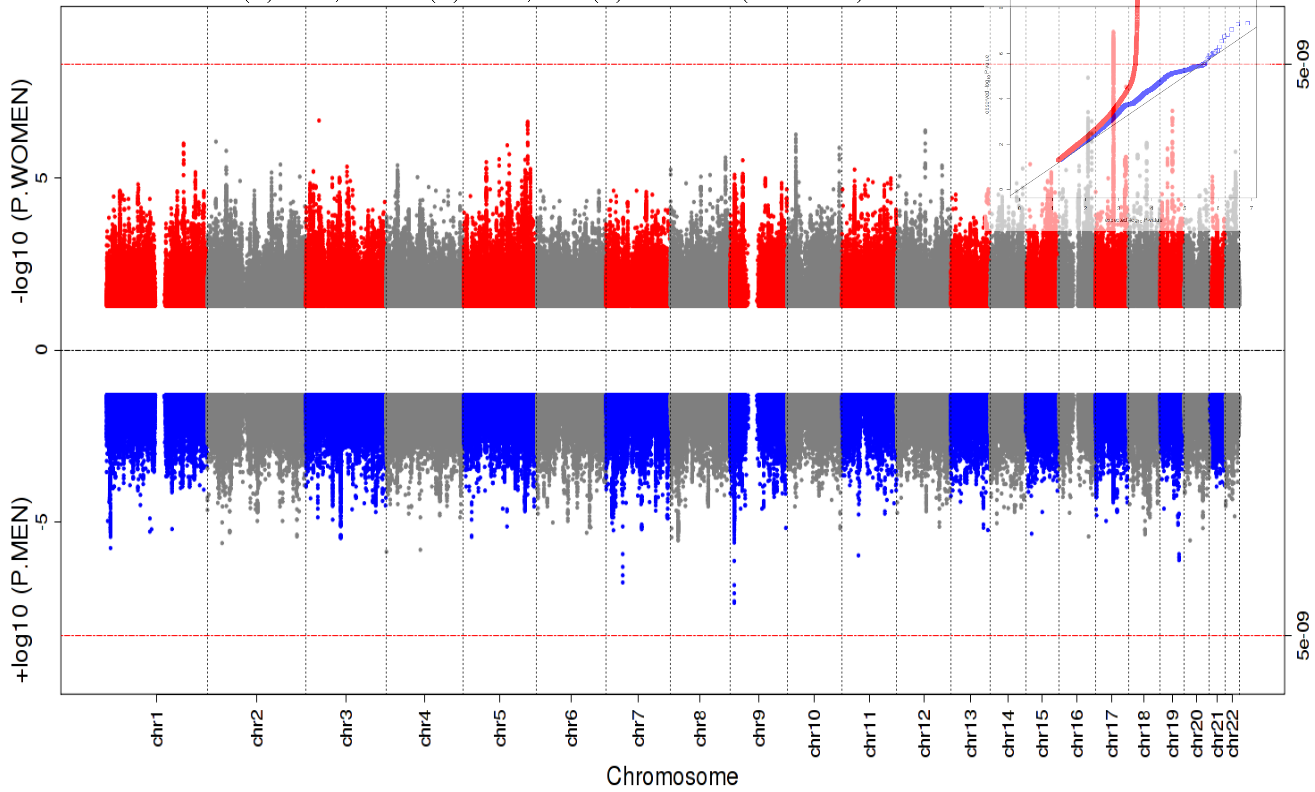

Sleep duration: λ (m) = 1.1, LD α (m) = 1.01, h^2 (m) = 0.2, R^2(5x10^{-7}hits m) = 0.26%
Rg = 0.96 λ (w)= 1.1, LD α (w) = 1.0, h^2 (w) = 0.19 R^2(5x10^{-7}hits f) = 0.15%

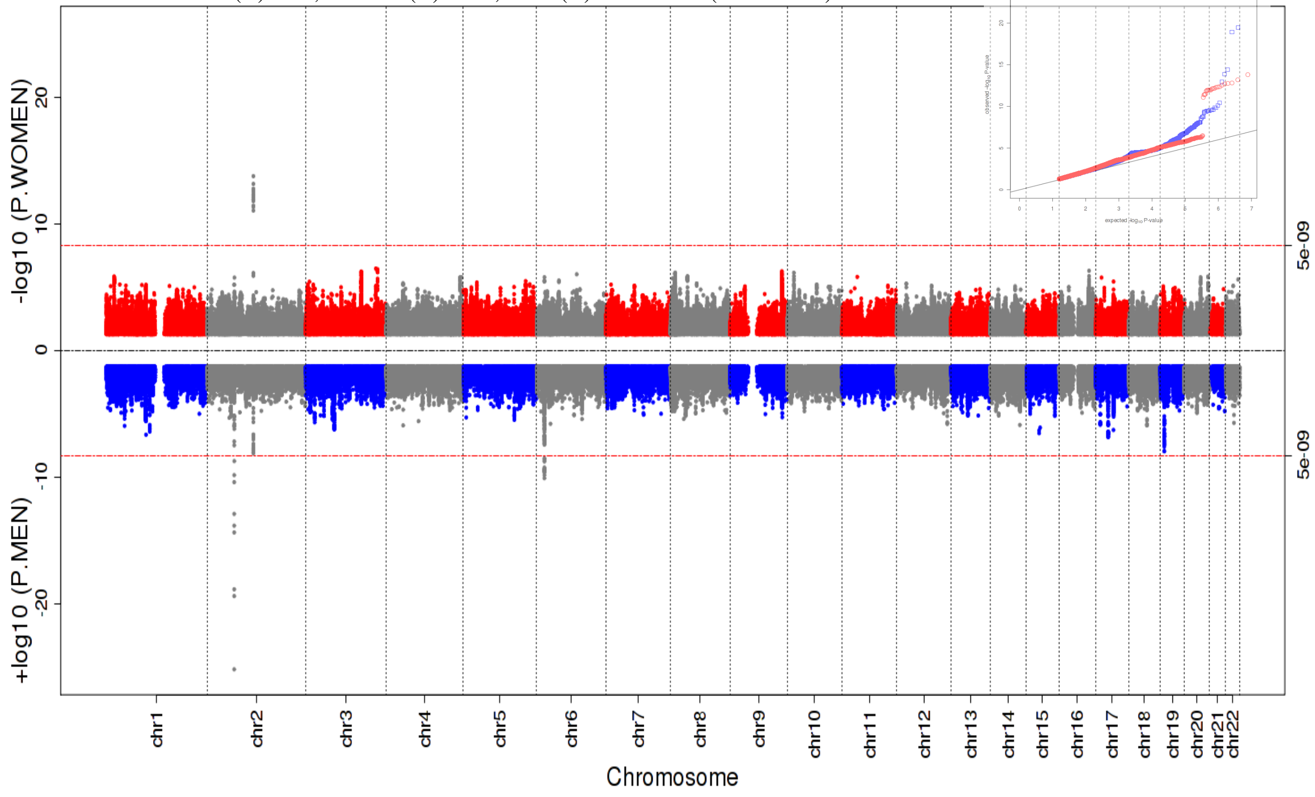

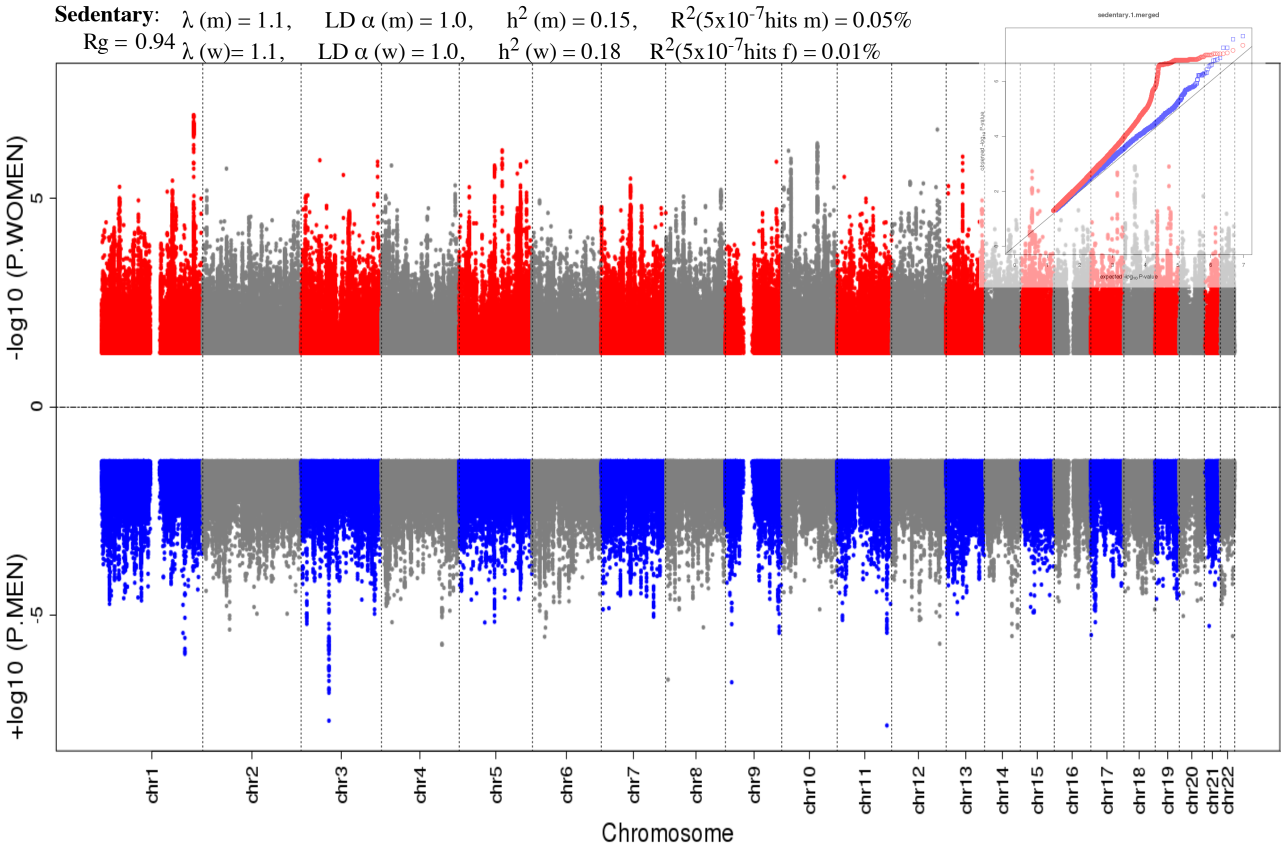
Sedentary: λ (m) = 1.1, LD α (m) = 1.0, h2 (m) = 0.15, R2(5x10-7hits m) = 0.05%
Rg = 0.94 λ (w)= 1.1, LD α (w) = 1.0, h2 (w) = 0.18 R2(5x10-7hits f) = 0.01%
sedentary.1.merged
-log10 (P.WOMEN)
+log10 (P.MEN)
chr1 chr2 chr3 chr4 chr5 chr6 chr7 chr8 chr9 chr10 chr11 chr12 chr13 chr14 chr15 chr16 chr17 chr18 chr19 chr20 chr21 chr22
Chromosome

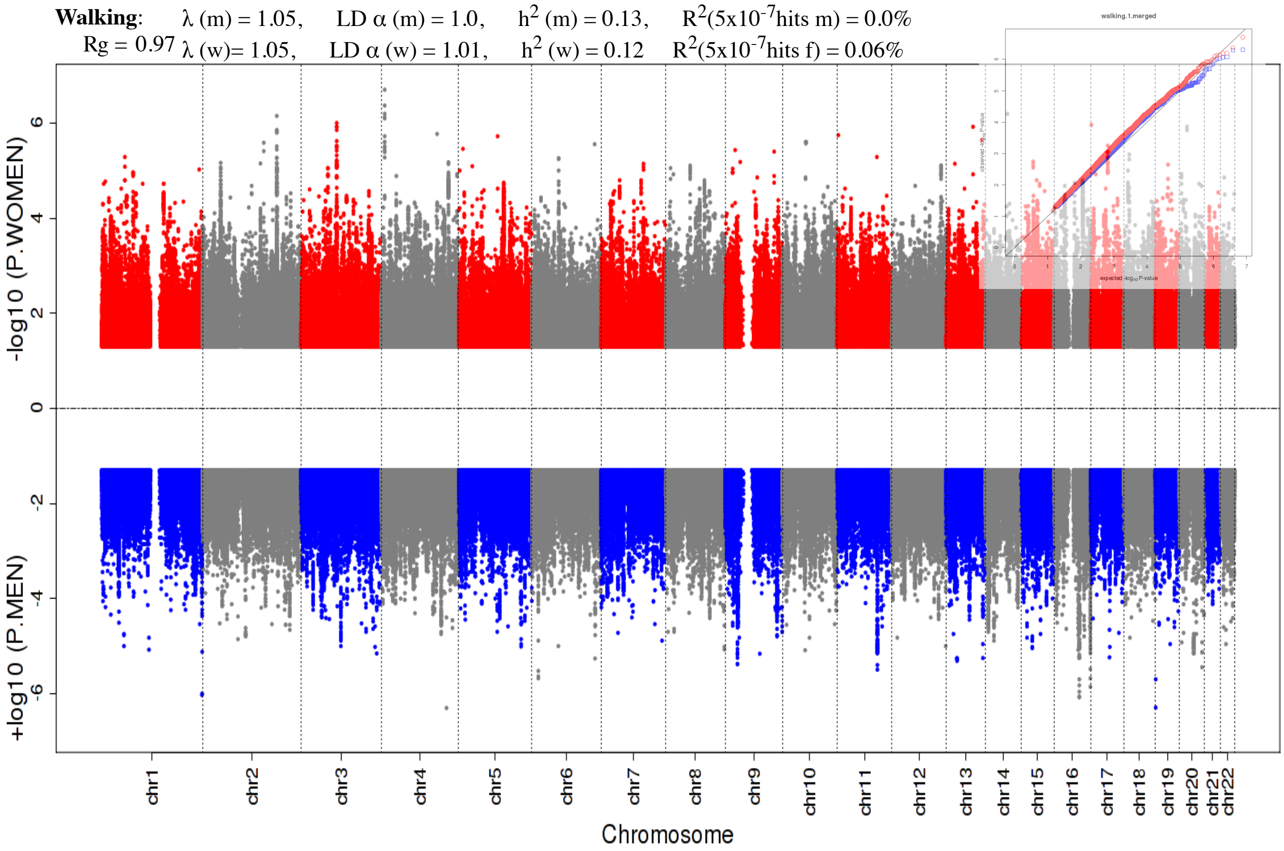
Walking: λ (m) = 1.05, LD α (m) = 1.0, h2 (m) = 0.13, R2(5x10-7hits m) = 0.0%
Rg = 0.97 λ (w)= 1.05, LD α (w) = 1.01, h2 (w) = 0.12 R2(5x10-7hits f) = 0.06%
walking.1.merged
-log10 (P.WOMEN)
+log10 (P.MEN)
chr1 chr2 chr3 chr4 chr5 chr6 chr7 chr8 chr9 chr10 chr11 chr12 chr13 chr14 chr15 chr16 chr17 chr18 chr19 chr20 chr21 chr22
Chromosome

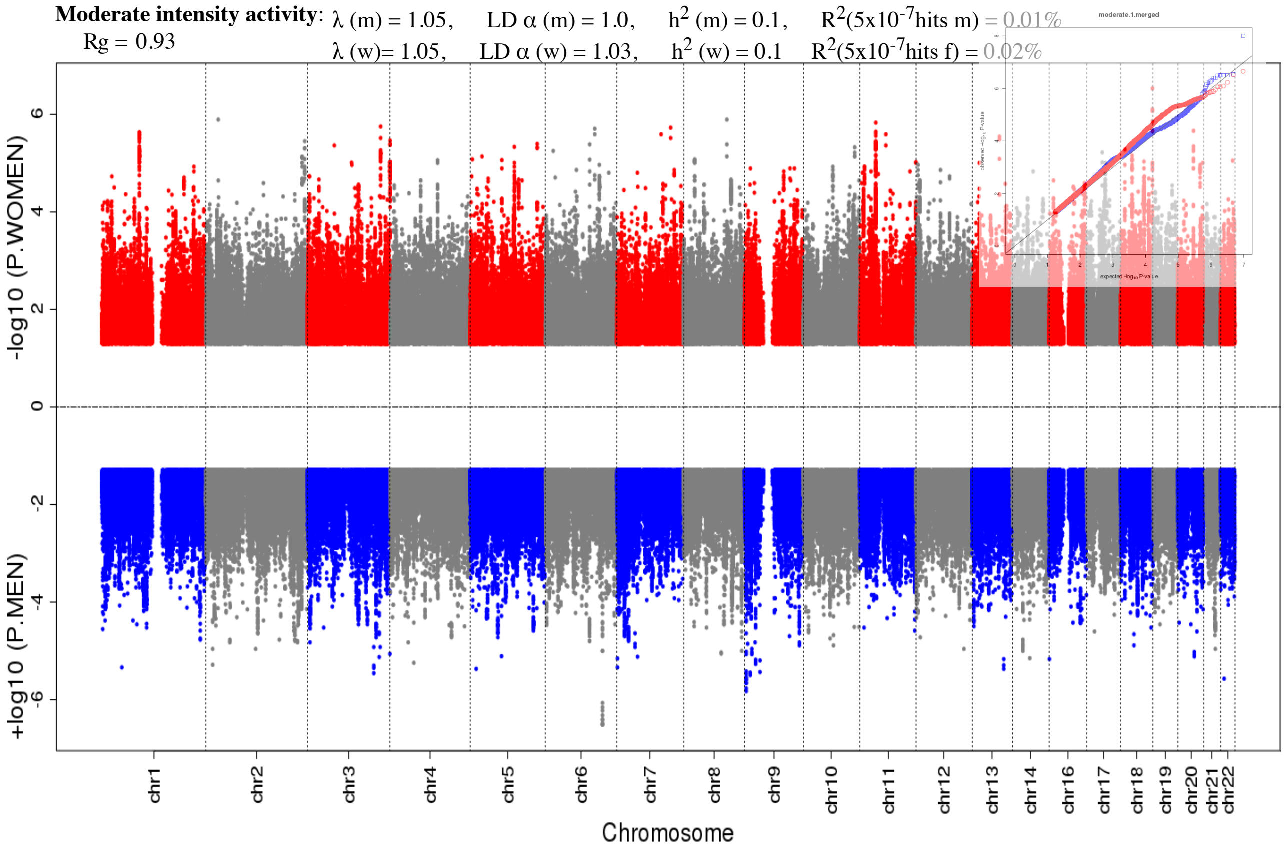
Moderate intensity activity: λ (m) = 1.05, LD α (m) = 1.0, h2 (m) = 0.1, R2(5x10-7hits m) = 0.01%
Rg = 0.93
λ (w)= 1.05, LD α (w) = 1.03, h2 (w) = 0.1 R2(5x10-7hits f) = 0.02%
moderate.1.merged
expected -log10 P-value
-log10 (P.WOMEN)
+log10 (P.MEN)
6
4
2
0
-2
-4
-6
chr1
chr2
chr3
chr4
chr5
chr6
chr7
chr8
chr9
chr10
chr11
chr12
chr13
chr14
chr16
chr17
chr18
chr19
chr20
chr21
chr22
Chromosome

Supplementary Figure 7 | Effect of p-value threshold for instrument selection on presence of detectable horizontal pleiotropy.
Boxplot represents 90 tests for five accelerometer exposure traits assessed against 18 outcomes in 278,374 UK Biobank participants who were not in the accelerometer discovery GWAS.

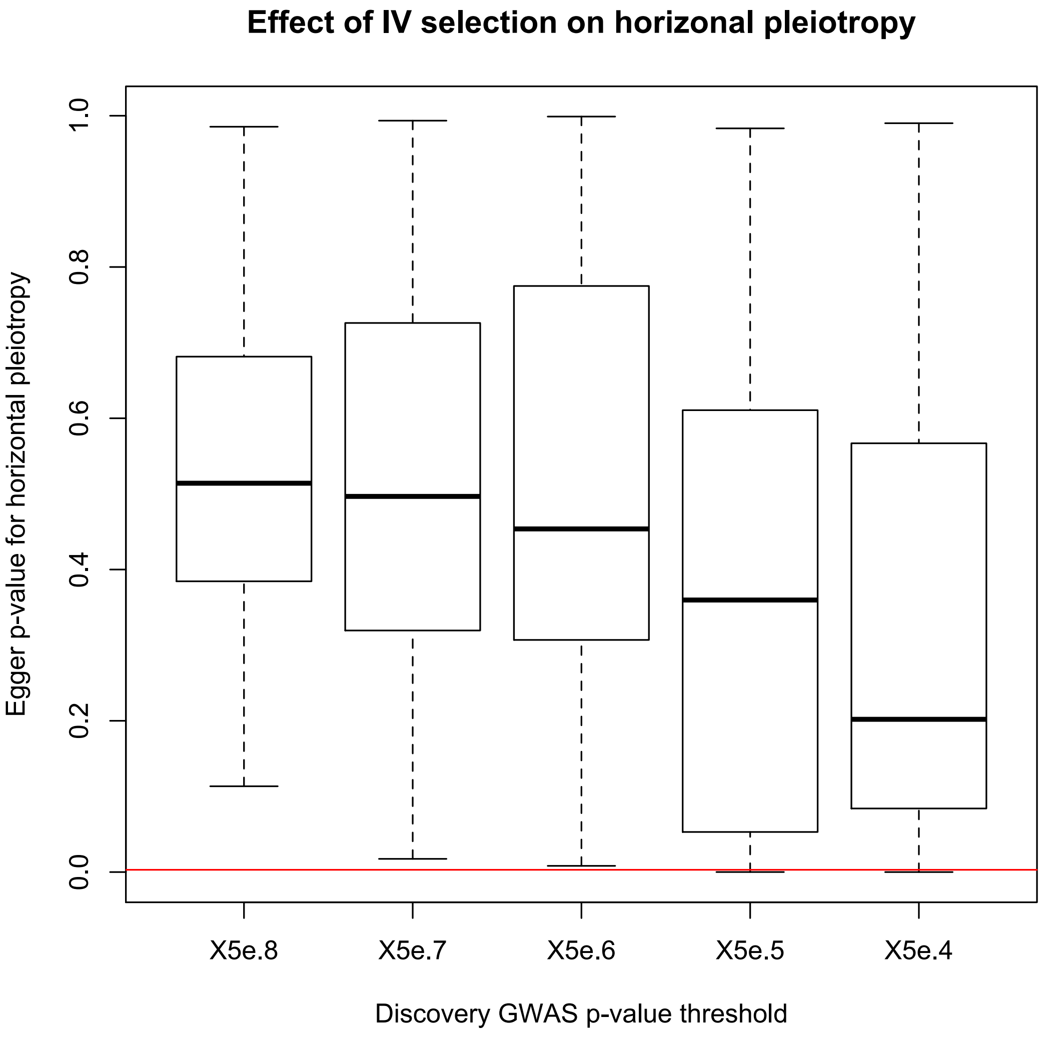

Supplementary Figure 8 | Mendelian randomisation leave-one-out and single-SNP-only sensitivity analyses for traits suggesting evidence of causality, colour-coded by p-value for instrument variable in discovery GWAS.

Leave one SNP out **Single SNP only**

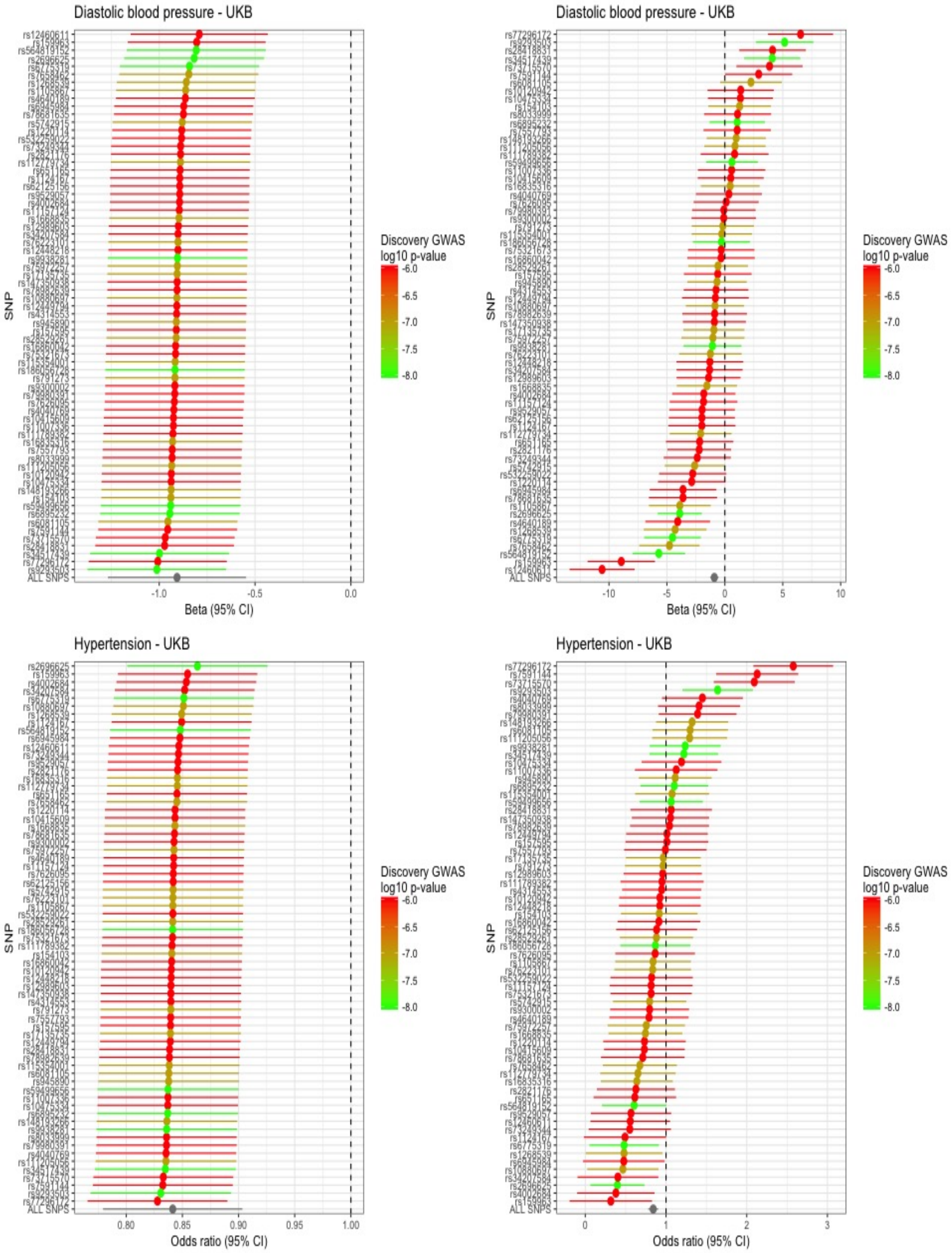

Leave one SNP out

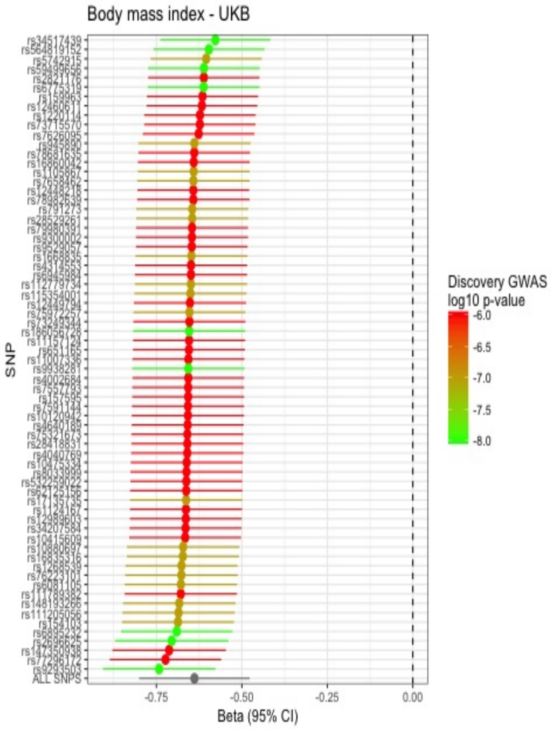

Single SNP only

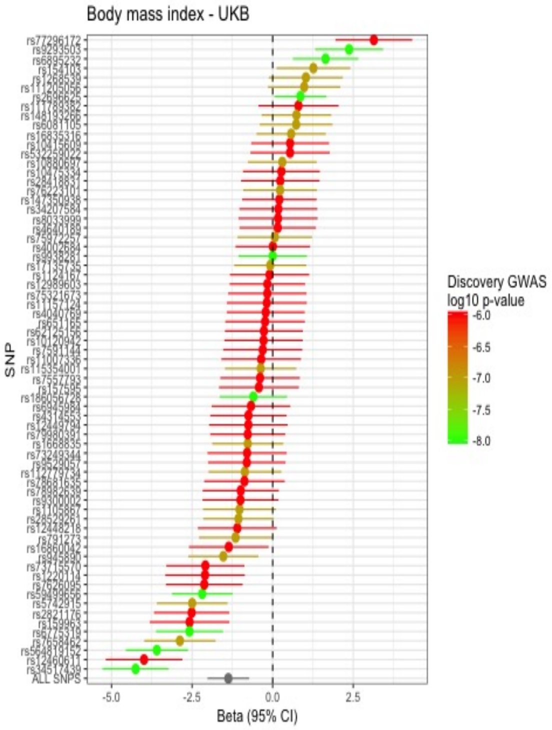

Supplementary Figure 9 | Tissue enrichment analysis using eQTL data from GTEx for accelerometer-measured traits in 91,105 UK Biobank participants.
Dotted line shows multiple testing threshold across phenotypes and tissue types.

Overall activity

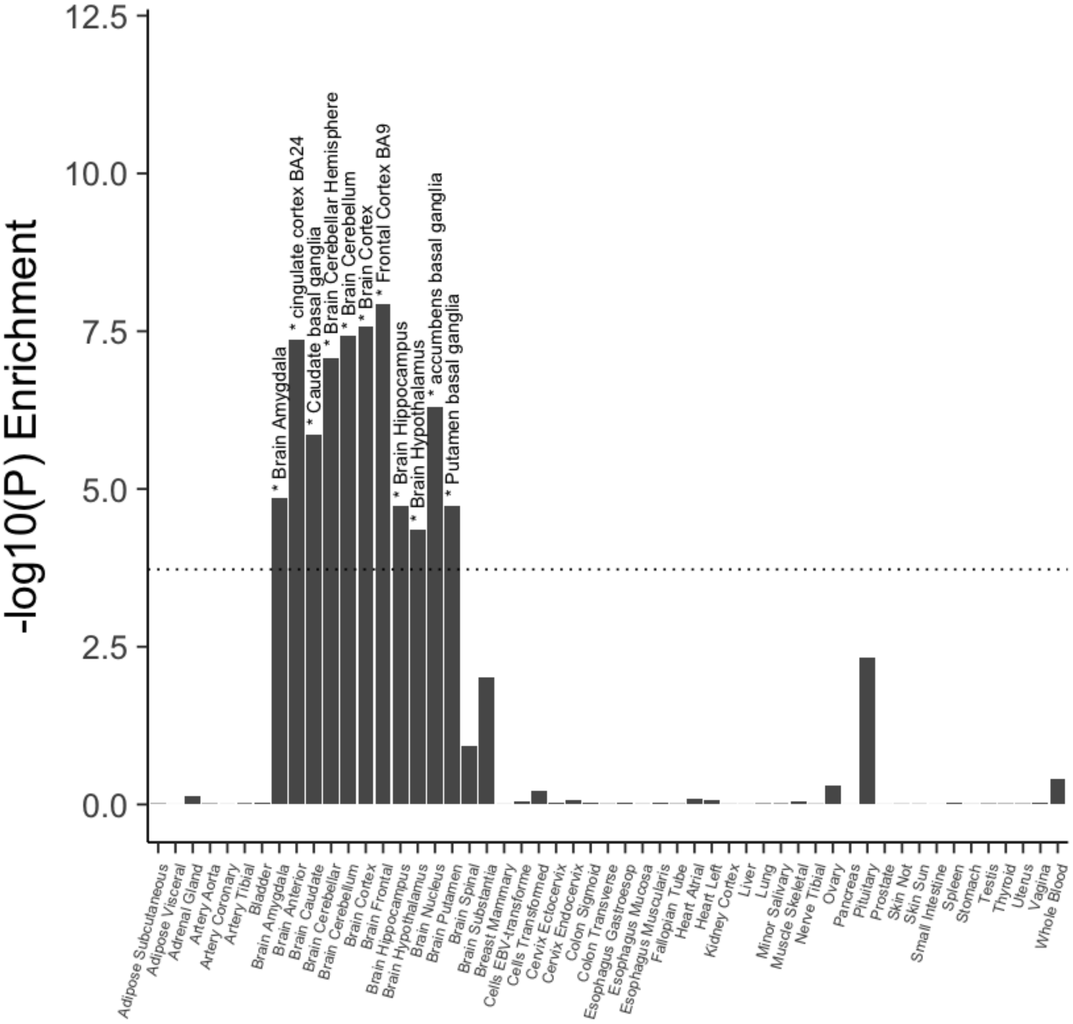

Sleep duration

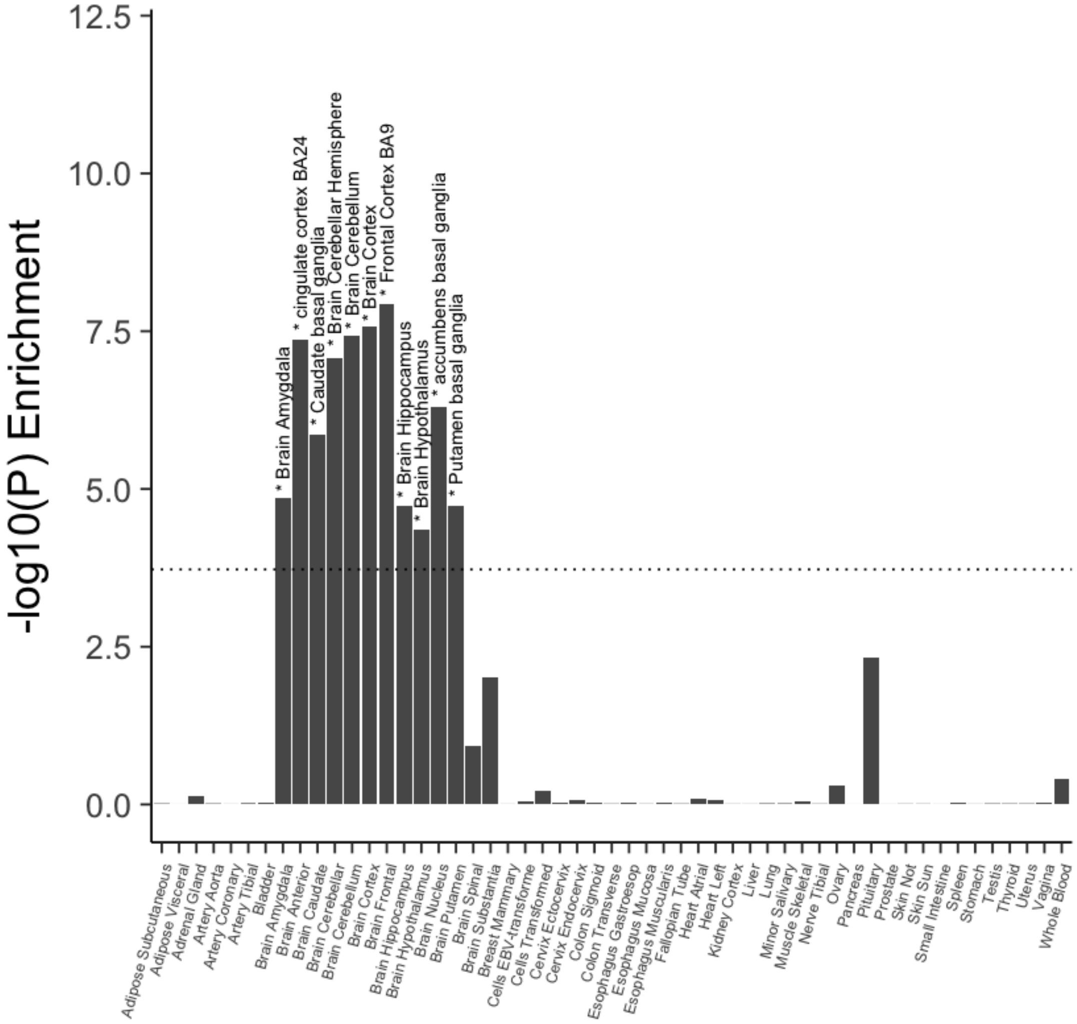

Sedentary behaviour – no significant associations.

Walking – no significant associations.

Moderate activity – no significant associations.

Supplementary Figure 10 | Gene set enrichment analysis of accelerometer-measured physical activity and sleep duration traits with other diseases.

Overall activity

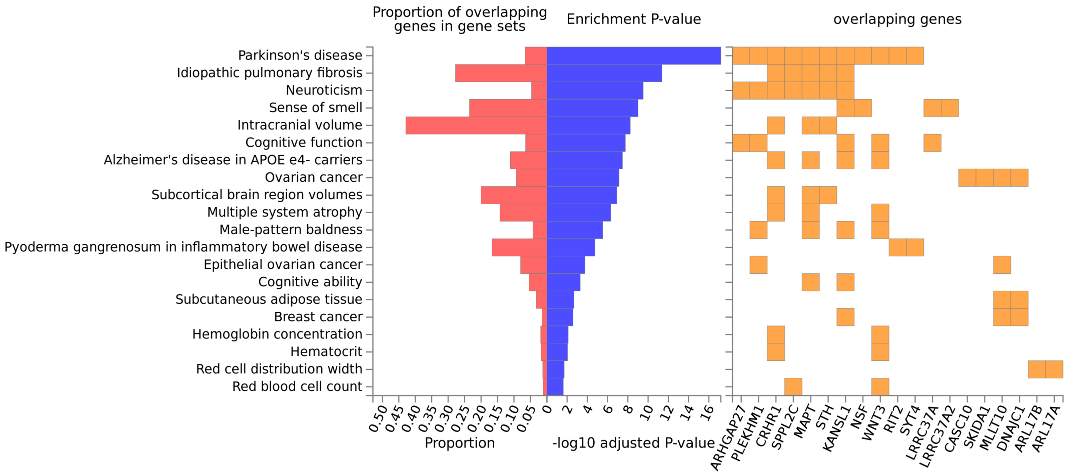

Sleep duration

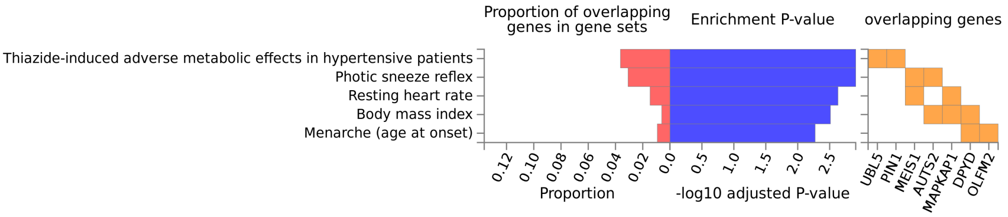

Sedentary

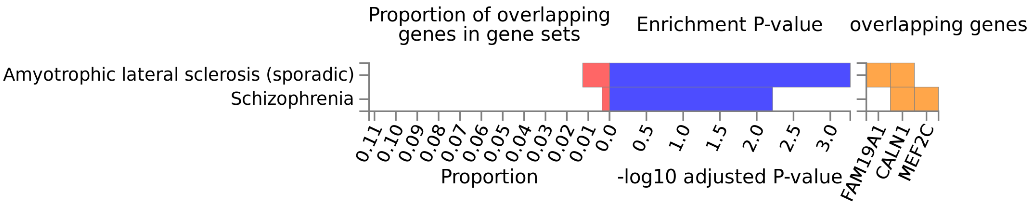

Walking – no significant loci on which to conduct analysis.

Moderate – no significant loci on which to conduct analysis.

Supplementary Figure 11 | Distribution of groundtruth activity states in training dataset (n=153) and predicted labels in UK Biobank accelerometer dataset (n=91,105).

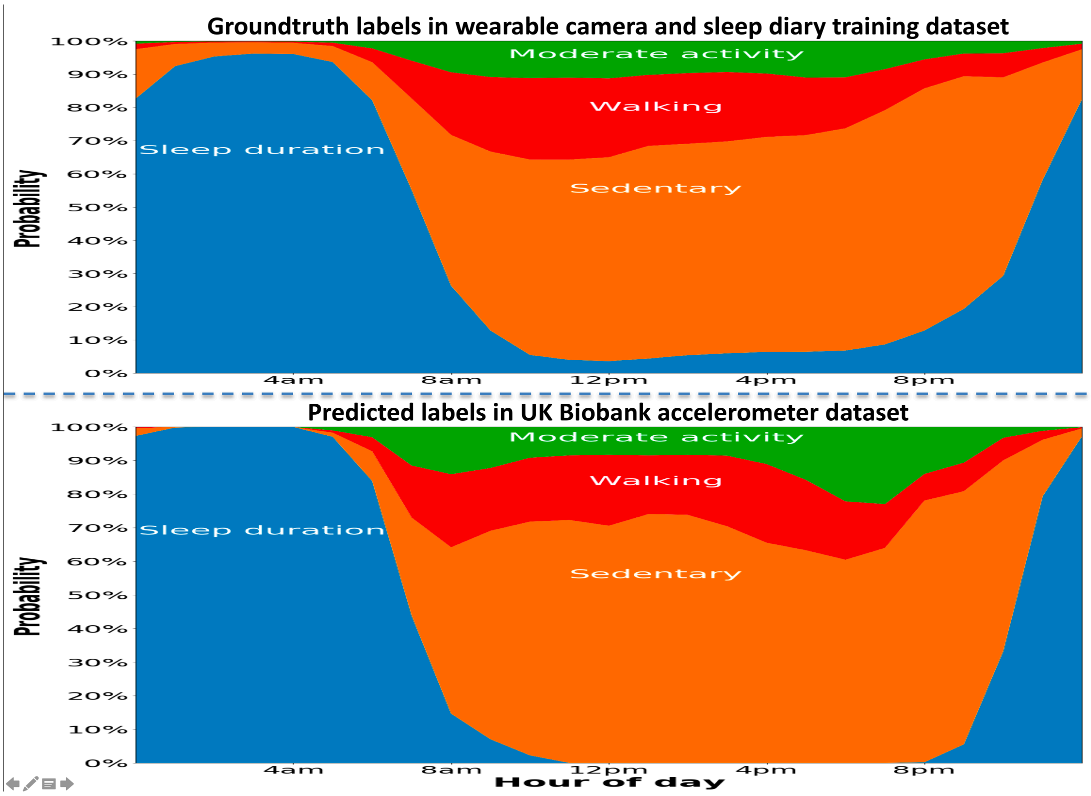

Supplementary Table 1 | Percentage of machine-learned physical activity behaviours and sleep duration automatically classified from wrist-worn accelerometer data.

Confusion matrix after leave-one-out validation on 188,355 labelled minutes of human activity in free-living environments: the CAPTURE-24 and ENERGY-24 studies 2014-2015 (n=153).

Prediction→ Ground truth↓	**Sleep duration**	**Sedentary**	**Tasks-light**	**Walking**	**Moderate activity**
**Sleep**	91%	8%	<1%	<1%	<1%
**Sedentary**	6%	81%	5%	3%	6%
**Tasks-light**	<1%	29%	25%	20%	26%
**Walking**	<1%	11%	15%	58%	16%
**Moderate**	<1%	12%	14%	15%	58%

Kappa agreement score = 0.68 **Overall classification accuracy = 79%**

Raw data for each 30-sec time window

Prediction→ Ground truth↓	**Sleep duration**	**Sedentary**	**Tasks-light**	**Walking**	**Moderate activity**
**Sleep**	154,099	12,985	652	427	358
**Sedentary**	6,782	97,899	6,230	3,576	6,938
**Tasks-light**	95	6,590	5,693	4,469	6,004
**Walking**	122	3,624	4,731	18,548	4,984
**Moderate**	94	2,682	3,153	3,311	12,664

Supplementary Table 2 | Fine-mapping of accelerometer measured physical activity and sleep duration loci in 91,105 UK Biobank participants.

TRAIT	LOCUS	CHR	SNP	P	# SNPs	# SNPS PREDICTED CAUSAL	# SNPS PLAUSIBLE CAUSAL	PLAUSIBLE SNPS DIST (KB)	MOST LIKELY CAUSAL SNP (PROB)	NEAREST GENE	DIST 2 ASSOC SNP (KB)
Overall activity	1	10	rs564819152	4.20E-09	4903	3	10	909.4	rs12247677 (0.97)	MIR4675	875.1
Overall activity	8	17	rs2696625	3.20E-12	6308	-	-	-	-	-	-
Overall activity	9	18	rs59499656	1.90E-09	6027	1	6	59.1	rs59499656 (0.24)	SYT4	0
Sleep duration	2	9	rs2416963	2.30E-10	4345	1	4	999.5	rs10819164 (0.08)	MVB12B	995.3
Sleep duration	3	7	rs2006810	3.90E-09	5967	1	5	50	rs2006810 (0.54)	AUTS2	0
Sleep duration	8	17	rs7502280	8.80E-11	6554	-	-	-	-	-	-
Sleep duration	10	2	rs62158170	5.80E-20	5662	1	6	5.4	rs62158170 (0.40)	PAX8-AS1	0
Sleep duration	11	2	rs113851554	3.10E-18	6813	1	1	0	rs113851554 (1.00)	MEIS1	0
Sleep duration	12	6	rs72828533	2.70E-13	8358	1	15	105.1	rs7765476 (0.11)	LOC101928519	3.8
Sleep duration	13	19	rs2303100	1.40E-10	6880	1	11	58.4	rs2303100 (0.11)	OLFM2	0
Sleep duration	14	1	rs75641275	2.20E-10	5759	1	4	235.1	rs75641275 (0.05)	DPYD	0
Sedentary	4	5	rs26579	2.60E-09	4198	1	7	12.3	rs26579 (0.09)	MEF2C-AS2	0
Sedentary	5	5	rs25981	3.00E-09	6395	1	9	23.7	rs25981 (0.26)	EFNA5	0
Sedentary	6	3	rs1858242	3.10E-09	6434	1	13	270.6	rs1858242 (0.19)	LOC105377146	0
Sedentary	7	7	rs34858520	4.20E-09	5491	1	0	-	-	-	-

Supplementary Note 1 | Physical activity and sleep duration, and their relationship with other traits

While physical activity and sleep duration are established risk factors for multiple diseases from observational epidemiology, the extent to which their underlying genetic architectures are shared with disease phenotypes is unknown[4]. First, we tested for genome-wide genetic correlations between our traits and those from publicly-available GWAS data in 832 phenotypes using LD score regression[15] on the LD-Hub web resource[27]. After adjusting for multiple testing ($p<1.2x10^{-5}$, accounting for 5 accelerometer phenotypes and 832 comparison traits), we found the movement phenotypes to correlate with 155 independent traits (**Supplementary Data 5**). In general, increases in overall activity and walking phenotypes were genetically correlated with improved health status in phenotypes relating to: BMI (r_g=-0.35, $p=2.8x10^{-58}$), body fat percentage (r_g=-0.43, $p=6.2x10^{-102}$), diabetes (r_g=-0.35, $p=2.2x10^{-25}$), cardiovascular disease (e.g. for myocardial infarction r_g=-0.25, $p=8.4x10^{-7}$), back-pain (r_g=-0.17, $p=4.5x10^{-6}$), hypertension (r_g=-0.24, $p=1.8x10^{-20}$), and HDL cholesterol (r_g=0.26, $p=5.9x10^{-12}$). Increases in sleep duration were genetically correlated with unfavourable status in phenotypes relating to: education (e.g. for age completed full time education r_g=-0.23, $p=6.1x10^{-12}$), fluid intelligence (r_g=-0.25, $p=1.6x10^{-15}$), and body fat percentage (r_g=0.12, $p=5.4x10^{-6}$). Increases in sedentary time were genetically correlated with increased fluid intelligence score (r_g=0.35, $p=1.5x10^{-24}$), but decreased health status (e.g. for body fat percentage r_g=0.27, $p=2.5x10^{-23}$) (**Supplementary Data 5**).

To examine whether the genome-wide significant loci identified in our analysis affect other traits, we used the Oxford Brain Imaging Genetics Server[25] (big.stats.ox.ac.uk) to perform a phenome-wide association study (PheWAS) on almost 4,000 traits in UK Biobank participants. After adjusting for multiple testing ($p<1.25x10^{-6}$, accounting

for 10 loci and 4,000 traits), we identified associations with 74 independent traits (**Supplementary Data 6**). These included anthropometric traits (n=39), self-reported brain imaging traits (n=11), and exercise traits (n=10) (complete results in **Supplementary Data 6**). We also extracted previously reported GWAS associations within 400kb and r^2>0.2 of accelerometer index SNPs from the NHGRI GWAS catalog[26]. Here, we identified 11 independent traits including cognitive and mental health outcomes (cognitive function, schizophrenia, autism, intelligence, and educational attainment) associated with accelerometer measured sleep duration (**Supplementary Data 7**).

Supplementary Note 2 | Mendelian Randomization decision tree

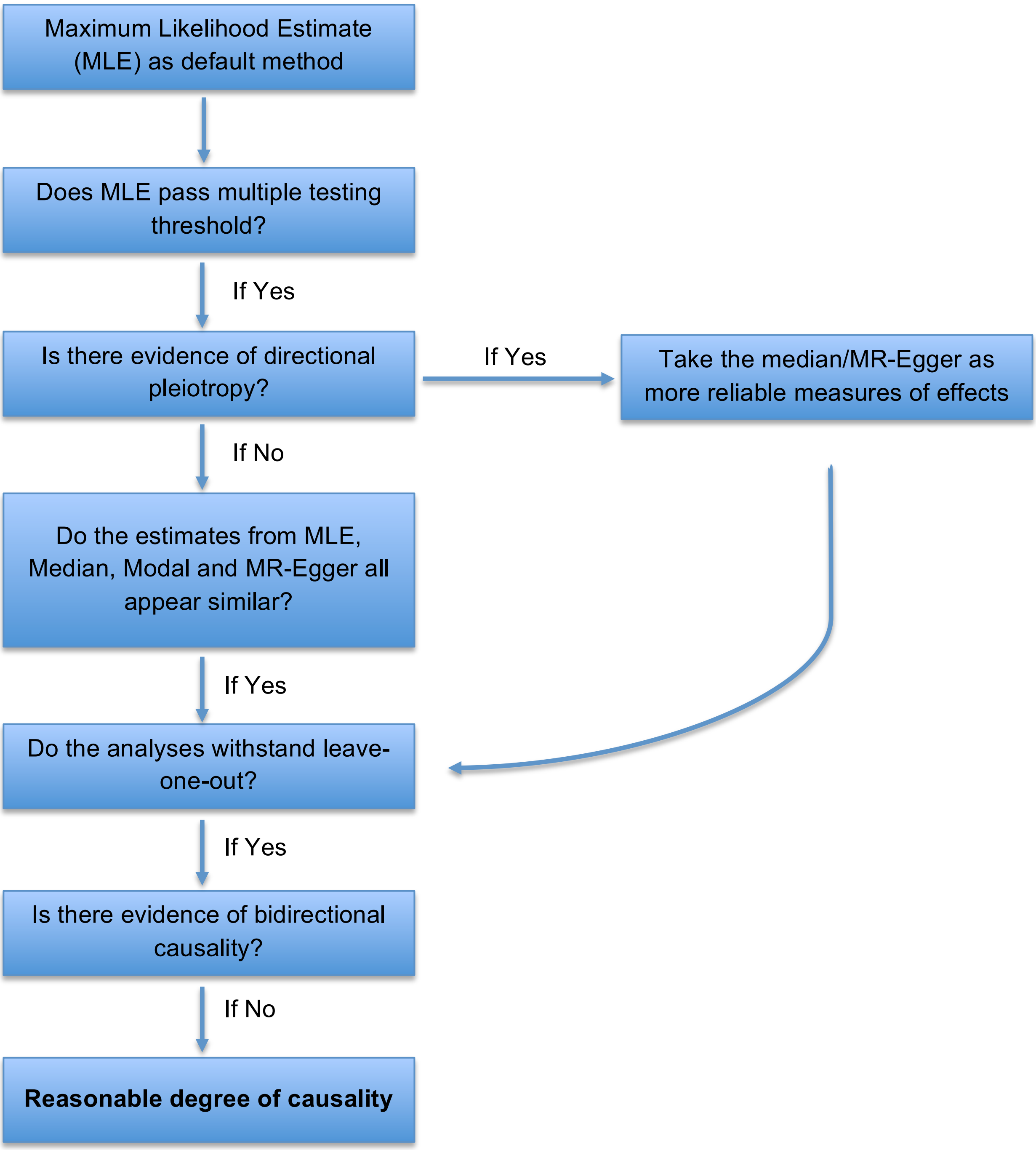

Supplement: Supplementary file 1 — Supplementary Information [file 41467_2018_7743_MOESM1_ESM.pdf]
