# Supplementary material for: GWAS identifies 14 loci for device-measured physical activity and sleep duration
Source: Nat Commun. 2018 Dec 10;9:5257. doi: 10.1038/s41467-018-07743-4 (PMC6288145; doi:10.1038/s41467-018-07743-4)
Supplement: Supplementary file 3 — Description of Additional Supplementary Files [file 41467_2018_7743_MOESM3_ESM.pdf]

**Supplementary Data 1 |** Phenotypes mapped from original image annotation and compendium of physical activities codes.

**Supplementary Data 2 |** Other suggestive ($5x10^{-8}$) loci associated with accelerometer-measured physical activity and sleep duration traits in 91,105 UK Biobank participants. *Beta and SE are in standard deviation units.*

**Supplementary Data 3 |** Results in this UK Biobank accelerometer cohort for genetic variants previously reported to be associated with traits related to physical activity and sleep duration in UK Biobank and other datasets. *Significant associations (p<2.3x10-4, accounting for 213 candidate loci) are highlighted in green. Beta and SE are in standard deviation units.*

**Supplementary Data 4 |** Significant genes associated with accelerometer-measured physical activity and sleep duration traits in 91,105 UK Biobank participants. *This is based on gene-based analysis by MAGMA integrated in FUMA where input SNPs were mapped by position to 18,232 protein-coding genes. Significant associations (p<5.48x10-7, accounting for 18,232-genes and 5 traits) did not find additional loci to those already identified in the SNP association analysis.*

**Supplementary Data 5 |** Significant genetic correlations for accelerometer-measured physical activity and sleep duration in 91,105 UK Biobank participants with other traits and diseases using LD score regression on the LD-Hub web resource.

**Supplementary Data 6 |** PheWAS results at P<1.25e-6 for genome-wide significant accelerometer measured physical activity and sleep duration loci in 91,105 UK Biobank participants.

**Supplementary Data 7 |** Reported associations of objectively measured physical activity and sleep duration SNPs identified in 91,105 UK Biobank participants with traits in the NHGRI GWAS catalog.

**Supplementary Data 8 |** Mendelian Randomization analysis of accelerometer-measured physical activity and sleep duration, and their association with disease outcomes. *This includes data from UK Biobank participants who were not in the accelerometer discovery dataset and other GWAS datasets available on MR-Base. SE and beta are in exposure SD units.*

**Supplementary Data 9 |** Meta analysis of Mendelian Randomization results for adiposity traits using GIANT and UK Biobank participants not in accelerometer discovery dataset. *SE and beta are in exposure SD units.*

**Supplementary Data 10 |** Effect of instrument variable selection by GWAS discovery threshold on Mendelian Randomization analysis outcomes. B*eta and SE are in exposure SD units.*

**Supplementary Data 11 |** Directionality, horizontal pleiotropy, and confounding sensitivity analyses for Mendelian Randomization associations suggesting evidence of causality from Supplementary Data 8, 9, and 10.

**Supplementary Data 12 |** Mendelian randomization bi-directional (and associated sensitivity) tests for traits suggesting evidence of causality.
